# The complete mitochondrial genome of the *Cyphastrea serailia*

**DOI:** 10.1080/23802359.2017.1347904

**Published:** 2017-07-11

**Authors:** Wentao Niu, Shuangen Yu, Bin Chen, Suharsono Suharsono

**Affiliations:** a Laboratory of Marine Biology and Ecology, Third Institute of Oceanography, State Oceanic Administration, Xiamen, China;; b Research Center for Oceanography LIPI, Jakarta, Indonesia

**Keywords:** Coral, mitogenome, phylogeny

## Abstract

In this study, the complete mitogenome sequence of stony coral, *Cyphastrea serailia* (Scleractinia), has been decoded for the first time by next-generation sequencing and genome assembly. The assembled mitogenome, consisting of 17,138 bp, has unique 13 protein-coding genes (PCGs), 3 transfer RNAs, and 2 ribosomal RNAs genes. The complete mitogenome of *Cyphastrea serailia* showed 97% identity to *Montastraea annularis*. The complete mitogenome provides essential and important DNA molecular data for further phylogenetic and evolutionary analysis for coral phylogeny.

The faviidae is one of the most important families of reef corals. *Cyphastrea serailia*, one of the members of faviidae family, is widely distributed throughout the Indo-Pacific (Swart et al. 2012), and it is common in most reef environments. The first establishment of *C. serailia* mitogenome is important for further evolutionary and phylogenetic analyses for stony coral.

Samples (voucher no. DYW14) of *Cyphastrea serailia* were collected from Daya Bay in Guangdong, China. We used next-generation sequencing to perform low-coverage whole genome sequencing according to the protocol (Niu et al. 2016). Initially, the raw next-generation sequencing reads were generated from HiSeq 2000 (Illumina, San Diego, CA). About 0.09% raw reads (3740 out of 4,024,910) were *de novo* assembled by using commercial software (Geneious V9, Auckland, New Zealand) to produce a single, circular form of complete mitogenome with about an average of 64× coverage.

The complete mitogenome of *Cyphastrea serailia* was 17,138 bp in size (GenBank KY094484) and its overall base composition is 25.0% for A, 13.0% for C, 20.5% for G and 41.4% for T, and have GC content of 33.5%, showing 97% identities to *Montastraea annularis* (GenBank AP008976). The protein-coding, rRNA and tRNA genes of *Cyphastrea serailia* mitogenome were predicted by using DOGMA (Wyman et al. 2004), ARWEN (Laslett and Canback 2008), MITOS (Bernt et al. 2013) tools and manually inspected. The complete mitogenome of *Cyphastrea serailia* includes unique 13 protein-coding genes (PCGs), 3 transfer RNA genes and 2 ribosomal RNA genes. All PCGs, tRNA and rRNA genes were encoded on the H-strand, except for one small 12S rDNA in the L-strand. Four PCGs of NAD4L, NAD5, COX2 and COX3 have intron insertion. The intron size ranges from 12 bp (for ND4L) to 11,002 bp (for ND5).

It is important to note that 6 PCGs started with ATG codon (ATP6, ATP8, CYTB, ND3, ND4 and ND6), 1 with ATT codon (COX1), 1 with GTG codon (COX3), 2 with TTG codon (ND1 and ND4L), 1 with ATA codon (ND2), 1 with CTG (COX2) and 1 with TTA codon (ND5). Nine of 13 PCGs are inferred to terminate with TAA (ATP6, ATP8, COX1, COX3, ND2, ND3, ND4L, ND6 and COX2), 4 PCGs with TAG (CYTB, ND1, ND4 and ND5). Among 13 PCGs, the longest one is ND5 gene (1,707 bp), whereas the shortest is ATP8 gene (198 bp).

To validate the phylogenetic position of *Cyphastrea serailia,* we used MEGA6 software (Tamura et al. 2013) to construct a Maximum likelihood tree (with 500 bootstrap replicates and Kimura 2-parameter model) containing complete mitogenomes of 12 species derived from Suborder of Faviina of Scleractinia. *Corallimorphus profundus* derived from Corallimorphidae was used as an outgroup for tree rooting. Result shows *Cyphastrea serailia* is closely related to *Montastraea franksi* with high bootstrap value supported (Figure 1). In conclusion, the complete mitogenome of the *Cyphastrea serailia* deduced in this study provides essential and important DNA molecular data for further phylogenetic and evolutionary analysis for stony coral phylogeny.

**Figure 1.**
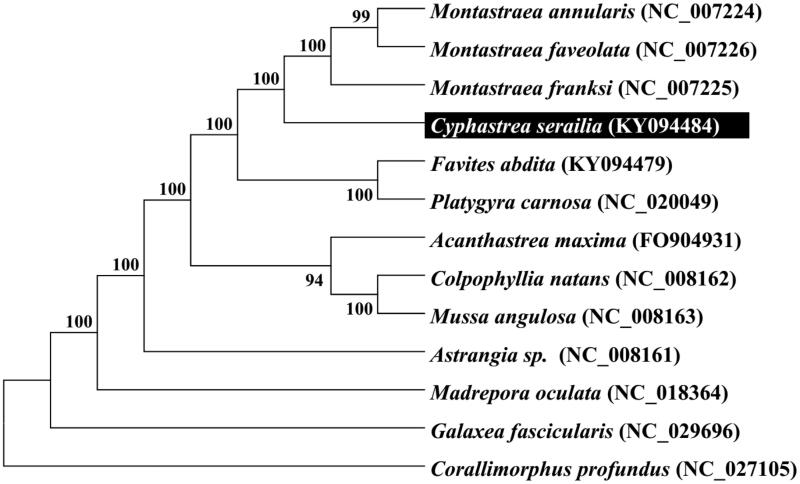
Molecular phylogeny of *Cyphastrea serailia* and related species in Scleractinia based on complete mitogenome. The complete mitogenomes is downloaded from GenBank and the phylogenic tree is constructed by maximum likelihood method with 500 bootstrap replicates. The gene’s accession number for tree construction is listed behind the species name.
